# Promyelocytic Leukemia Proteins Regulate Fanconi Anemia Gene Expression

**DOI:** 10.3390/ijms22157782

**Published:** 2021-07-21

**Authors:** Anudari Munkhjargal, Myung-Jin Kim, Da-Yeon Kim, Young-Jun Jeon, Young-Hoon Kee, Lark-Kyun Kim, Yong-Hwan Kim

**Affiliations:** 1 Department of Biological Sciences, Research Institute of Women’s Health, College of Natural Sciences, Sookmyung Women’s University, Seoul 04310, Korea; anukmunkhu@gmail.com (A.M.); pisces13th@sookmyung.ac.kr (M.-J.K.); dayeon_0630@sookmyung.ac.kr (D.-Y.K.); 2 Department of Integrative Biotechnology, Sungkyunkwan University, Suwon 16419, Korea; jeon2020@skku.edu; 3 Department of New Biology, Daegu Gyeongbuk Institute of Science and Technology, Daegu 42988, Korea; ykee@dgist.ac.kr; 4 Severance Biomedical Science Institute, Graduate School of Medical Science, Brain Korea 21 Project, Gangnam Severance Hospital, Yonsei University College of Medicine, Seoul 06230, Korea

**Keywords:** PML nuclear body, Fanconi anemia, interstrand DNA crosslink, CHK1 inhibitors

## Abstract

Promyelocytic leukemia (PML) protein is the core component of subnuclear structures called PML nuclear bodies that are known to play important roles in cell survival, DNA damage responses, and DNA repair. Fanconi anemia (FA) proteins are required for repairing interstrand DNA crosslinks (ICLs). Here we report a novel role of PML proteins, regulating the ICL repair pathway. We found that depletion of the PML protein led to the significant reduction of damage-induced *FANCD2* mono-ubiquitination and *FANCD2* foci formation. Consistently, the cells treated with siRNA against PML showed enhanced sensitivity to a crosslinking agent, mitomycin C. Further studies showed that depletion of PML reduced the protein expression of *FANCA*, *FANCG*, and *FANCD2* via reduced transcriptional activity. Interestingly, we observed that damage-induced CHK1 phosphorylation was severely impaired in cells with depleted PML, and we demonstrated that CHK1 regulates *FANCA*, *FANCG*, and *FANCD2* transcription. Finally, we showed that inhibition of CHK1 phosphorylation further sensitized cancer cells to mitomycin C. Taken together, these findings suggest that the PML is critical for damage-induced CHK1 phosphorylation, which is important for FA gene expression and for repairing ICLs.

## 1. Introduction

The promyelocytic leukemia (*PML*) gene was first discovered as a fusion partner of retinoic acid receptor α (RARα) in acute promyelocytic leukemia [1]. *PML* protein is the core component of multifaceted subnuclear structures known as *PML* nuclear bodies (*PML* NBs) that are implicated in the regulation of cellular functions including cell proliferation, apoptosis, senescence, tumor suppression, DNA repair, and DNA damage responses [2,3,4,5,6]. *PML* NBs of subnuclear spherical structure ranging from 0.1 to 1 μm in diameter contain diverse annotated domains, allowing them to interact with a variety of binding partners and facilitates their functions [2,7,8]. Based on more than a decade of studies, *PML* NBs are functionally associated with over 160 proteins directly and indirectly [3,9]. Seven *PML* isoforms, I~VIIb, have been characterized by their C-terminal ends, which determine their specific functions [10]. It was reported that the C-terminal of *PML* IV interacts with p53, which leads to the recruitment of p53 to *PML* NBs [11]. *PML* is also important for damage-induced MRE11, BRCA1, and RPA foci formation, which is essential for DNA double-strand break repair [12].

Fanconi anemia (FA) is a rare recessive genetic disorder characterized by bone marrow failure, congenital malformations, and cancer predisposition [13,14]. Under cellular study, cells obtained from FA patients are hypersensitive in response to mitomycin C (MMC), which induces interstrand DNA crosslinks (ICLs) [15]. To date, researchers have identified loss-of-function mutations of 22 genes in FA patients. Among them, nine FA proteins, FANCM, -L, -G, -A, -E, -C, -B, and -F form a FA core complex with FA-associated proteins such as FAAP20, FAAP24, FAAP100, MHF1, and MHF2 [3,13,14,16]. The FA core complex, as a ubiquitin E3 ligase, facilitates sensing DNA lesions and mono-ubiquitinates *FANCD2* and FANCI. The mono-ubiquitinated *FANCD2* and FANCI form a heterodimer ID complex by binding to each other, and the mono-ubiquitination of *FANCD2* and FANCI is widely accepted as a biomarker for the presence of ICLs. Recruitment of ID complex to the sites of DNA lesions is critical for the FA pathway to repair ICLs including incision, unhooking, error prone translesion synthesis, and DNA double-strand break (DSB) repair [17,18]. The ID complex is important for regulating the functions of downstream factors of the FA pathway including FANCD1, -N, -J, -S, and -O [19,20]. Mono-ubiquitinated *FANCD2* is recruited to DNA lesions and promotes DNA repair by homologous recombination [21,22].

CHK1 was first known as a Ser/Thr protein kinase regulating G2/M phase transition to DNA damage in fission yeast [23]. Human and *Xenopus* CHK1 were phosphorylated in response to DNA damage [24,25]. CHK1 has a key role in delivering checkpoint signals received from the ATM and ATR, which phosphorylate CHK1 and CHK2 [26]. Unlike CHK2, which is expressed throughout the cell cycle, CHK1 is mainly involved in the S and G2 phase to arrest cell cycle progression in response to DNA damage [27]. It has been well recognized that cancer cells with p53 mutations rely heavily on CHK1 functions for survival [28], which is supported by the findings showing that CHK1 is overexpressed in several human tumors [29,30,31]. Therefore, CHK1 has been a potential target for cancer therapy and, as a result, CHK1 inhibitors have been developed. Particularly, it was reported that enhanced CHK1 activity is correlated with cancer resistance, and thus CHK1 inhibitors showed synergistic cancer therapy efficacy together with chemotherapeutics including gemcitabine and cisplatin [28].

Here, we examined the mono-ubiquitination and foci formation of *FANCD2* in the absence of *PML* NBs to address the functional roles of *PML* NBs in the FA pathway. Depletion of *PML* in U2OS and HeLa cells resulted in loss of foci formation and mono-ubiquitination of *FANCD2*. Interestingly, we found that damage-induced CHK1 phosphorylation is severely impaired in cells depleted with *PML*. In addition, we showed that CHK1 plays a critical role in Fanconi anemia gene expression, demonstrating that *PML* regulates FA gene expression by promoting damage-induced CHK1 phosphorylation. These findings will be informative for better understanding the molecular basis for the synergistic chemotherapeutic effects of combined treatment with CHK1 inhibitors and crosslinking agents.

## 2. Results

### 2.1. Depletion of PML Causes Impairment of Damage-Induced FANCD2 Mono-Ubiquitination and FANCD2 Foci Formation

*PML* NBs are implicated in diverse DNA damage responses and DNA repair pathways. In this study, we aimed to understand the roles of *PML* NBs in the Fanconi anemia pathway, which is critical for repairing ICL damage. To this end, we depleted *PML* proteins and tested if *FANCD2* was mono-ubiquitinated upon MMC treatment. As a control, ATR protein was depleted because it is required for damage-induced *FANCD2* mono-ubiquitination [32].

**Figure 1:**
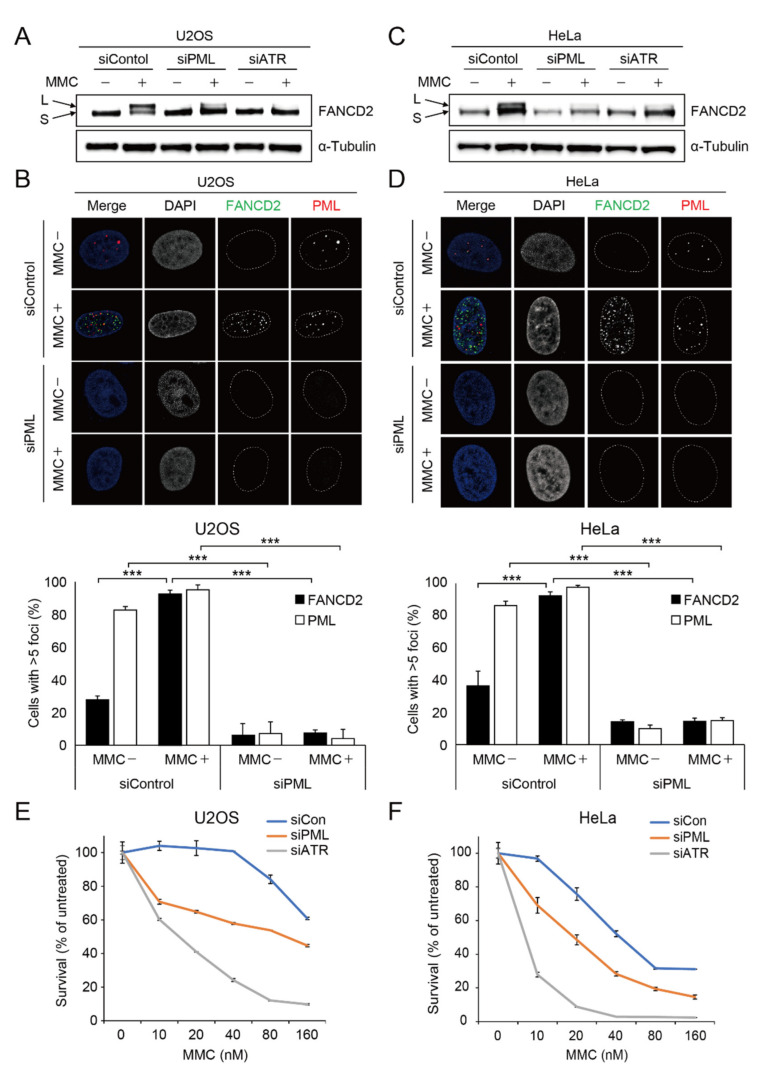
*PML* is required for *FANCD2* mono-ubiquitination and foci formation in response to genotoxic agents inducing interstrand DNA crosslinks. (**A**,**C**) U2OS and HeLa cells were transfected with siControl, si*PML*, or siATR and treated with 1 μM mitomycin C (MMC) for 24 h. *FANCD2* protein expression was determined though Western blotting of cell lysates from U2OS and HeLa. Long form (L) of *FANCD2* represents mono-ubiquitination and short form (S) is non-ubiquitinated *FANCD2*. (**B**,**D**) *FANCD2* foci formation in U2OS and HeLa cells treated with siControl or si*PML* under 1 μM MMC exposure for one day. Representative immunofluorescence images and their quantification. Over 100 nuclei were counted for statistics. Data represent the mean ± SD from three independent experiments (*** *p* < 0.001). (**E**,**F**) Proliferation level of *PML*- or ATR-deficient U2OS and HeLa cells treated with indicated doses of MMC for four or five days.

Interestingly, we found that loss of *PML* protein severely impaired *FANCD2* mono-ubiquitination in U2OS cells (Figure 1A). Consistently, damage-induced *FANCD2* foci formation was abrogated in the U2OS cells depleted with *PML* proteins (Figure 1B). It was reported that the localization and functions of *PML* NBs in cells that activate alternative lengthening of telomeres (ALT) are different from the processes in non-ALT cells [33,34]. Whereas U2OS cells elongate the telomeres by ALT, HeLa cells extend the telomeres through telomerase. Therefore, to test if *PML* NBs also affected *FANCD2* mono-ubiquitination and damage-induced foci formation in non-ALT cells, we depleted *PML* protein in HeLa cells, followed by MMC treatment. As shown in Figure 1C–D, HeLa cells treated with siRNA against the *PML* protein showed impaired *FANCD2* mono-ubiquitination and failed to form damage-induced *FANCD2* foci, which suggests that *PML* NBs regulate *FANCD2* functions in both ALT and non-ALT cells. Because *FANCD2* mono-ubiquitination and foci formation are critical for resistance to the genotoxic agents that cause ICLs, we tested if *PML* depletion led to hypersensitivity to MMC. As shown in Figure 1E,F, U2OS or HeLa cells depleted with ATR, as a control, showed higher sensitivity to MMC. We found that depletion of *PML* NBs in both cells also led to greater MMC sensitivity compared to control cells. Taken together, these findings suggest that *PML* NBs plays an important role in repairing interstrand DNA crosslink damage through promoting the Fanconi anemia pathway.

### 2.2. Damage-Induced FANCA Foci Formation Is Impaired in Cells with PML Depletion

**Figure 2:**
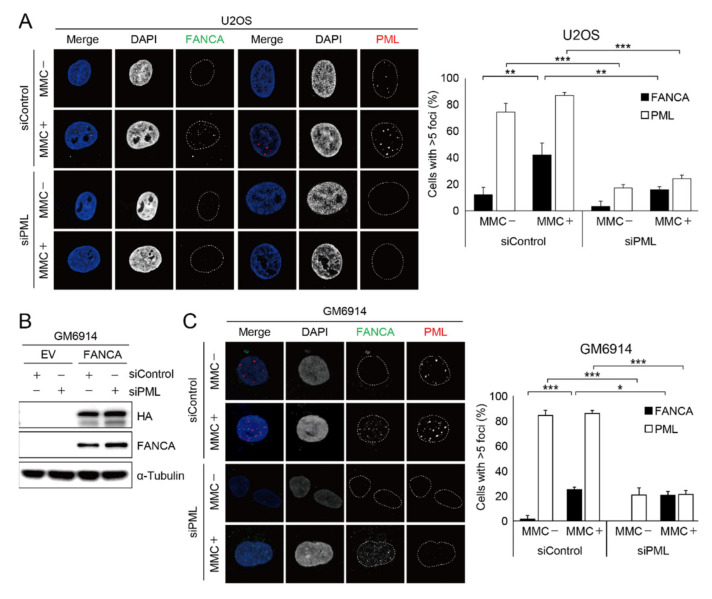
*PML* induces *FANCA* recruitment at the sites of DNA damage. (**A**) U2OS cells transfected with siControl or si*PML* were treated with 1 μM MMC. The next day, cells were immunostained with the endogenous anti-*FANCA* antibody. Representative images and their quantification are shown. Over 40 nuclei were counted for statistics. Values are expressed as mean ± SD from three independent experiments (*** *p* < 0.001; ** *p* < 0.01). (**B**) Depletion of *PML* in GM6914 expressing empty vector or HA-tagged *FANCA*. HA and *FANCA* protein expression was determined by Western blot assay. (**C**) After *PML* depletion, GM6914/*FANCA* cell lines were treated with 1 μM MMC for 24 h and immunostained with the anti-HA and anti-*PML* antibodies. Representative immunofluorescence images and their quantification are shown. Over 40 nuclei were counted for statistics. Data represent the mean ± SD from three independent experiments (*** *p* < 0.001; * *p* < 0.05).

Recruitment of the Fanconi anemia core complex to the sites of DNA damage is crucial for *FANCD2* mono-ubiquitination during ICL repair. To gain insight into the molecular basis for the observation that loss of *PML* proteins impairs the damage-induced foci formation and mono-ubiquitination of *FANCD2*, we first tested if the FA core complex moves to sites of DNA damage. As shown in Supplementary Figure S1, damage-induced *FANCA* foci are nicely co-localized with damage markers such as γH2AX and *FANCD2* in U2OS cells treated with MMC, which induces ICL damage. In the same setting, we depleted *PML* protein and tested if damage-induced *FANCA* foci are formed. Interestingly, we found that damage-induced *FANCA* foci formation was severely impaired in the U2OS cells (Figure 2A). *PML* proteins are essential components of *PML* nuclear bodies. *PML* NBs are subnuclear organelles that regulate the stability, functions, and localization of various proteins. Therefore, we asked if the *PML* NBs directly regulate *FANCA* recruitment to the sites of DNA damage or play a role in *FANCA* gene expression. To this end, we took advantage of the Fanconi anemia patient cell line, GM6914 [35], which does not express endogenous *FANCA* protein and GM6914 cells complemented with either empty vector (GM6914/EV) or HA-tagged wildtype *FANCA* (GM6914/*FANCA*). As shown in the Figure 2B,C, we found that exogenous *FANCA* expression under a CMV promoter was not reduced and damage-induced *FANCA* foci formation was normal upon siRNA-mediated *PML* depletion in the GM6914/*FANCA* cells. These findings imply that protein stability of the FA core complex is not affected by the *PML* NBs, and that *PML* NBs regulate the expression of Fanconi anemia proteins at the transcriptional level.

### 2.3. PML Regulates FANCA, FANCG, and FANCD2 Gene Expression

**Figure 3:**
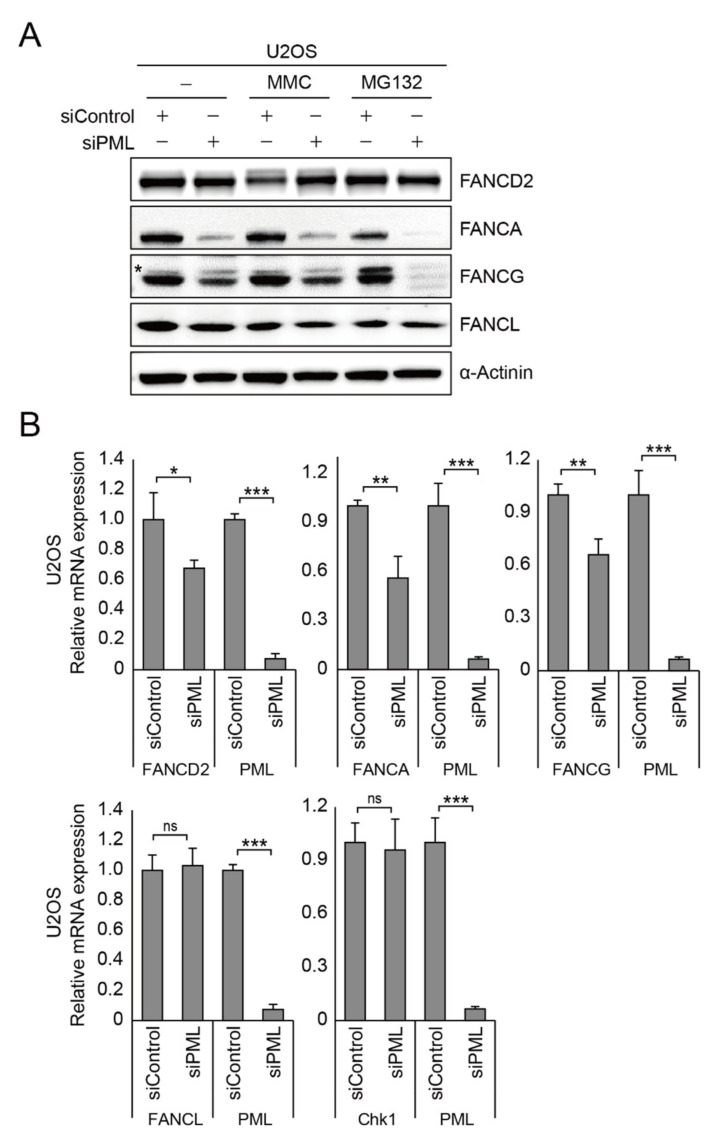
*PML* NBs control gene expression of Fanconi anemia core complex. (**A**) After depletion of *PML*, U2OS cells were treated with 1 μM MMC or 10 μM MG132 for 24 h. Expression of *FANCD2* and FA core proteins including *FANCA*, *FANCG*, and *FANCL* was analyzed by immunoblotting assay. The asterisk indicates a cross-reactive artifact. (**B**) Relative mRNA expression of *FANCD2*, *FANCA*, *FANCG*, *FANCL*, *Chk1*, and *PML* was determined by real-time quantitative PCR in the U2OS cells depleted with *PML*. Data represent the mean ± SD from three independent experiments (*** *p* < 0.001; ** *p* < 0.01; * *p* < 0.05; ns, not significant (*p* > 0.05)).

The findings described above suggest that *PML* proteins might be implicated in Fanconi anemia gene expression. To test if *PML* proteins are important for expression of Fanconi anemia proteins, we depleted *PML* in U2OS cells and performed immunoblot analysis with and without MMC treatment. As shown in Figure 3A and Supplementary Figure S2A, we found that *FANCA* and *FANCG* protein level were lower in the cells depleted *PML* protein. However, *FANCL* expression level was not reduced, suggesting that not all FA protein gene expression is regulated by *PML*. In addition, to see if the FA core complex is one of the targets for ubiquitin-dependent proteasomal degradation, we treated the cells with MG132 after inducing DNA damage, but we found that there was no protein stability recovery upon MG132 treatment, indicating that *PML* might regulate expression of the FA core complex at the transcriptional level (Figure 3A). Indeed, we found significantly less *FANCA*, *FANCG*, and *FANCD2* at the transcriptional level in the absence of *PML* proteins, measured by real-time quantitative PCR (Figure 3B). Taken together, these findings suggest that *PML* regulates the gene expression of *FANCA*, *FANCG*, and *FANCD2*.

### 2.4. PML Promotes CHK1 Phosphorylation in Response to DNA Damage

**Figure 4:**
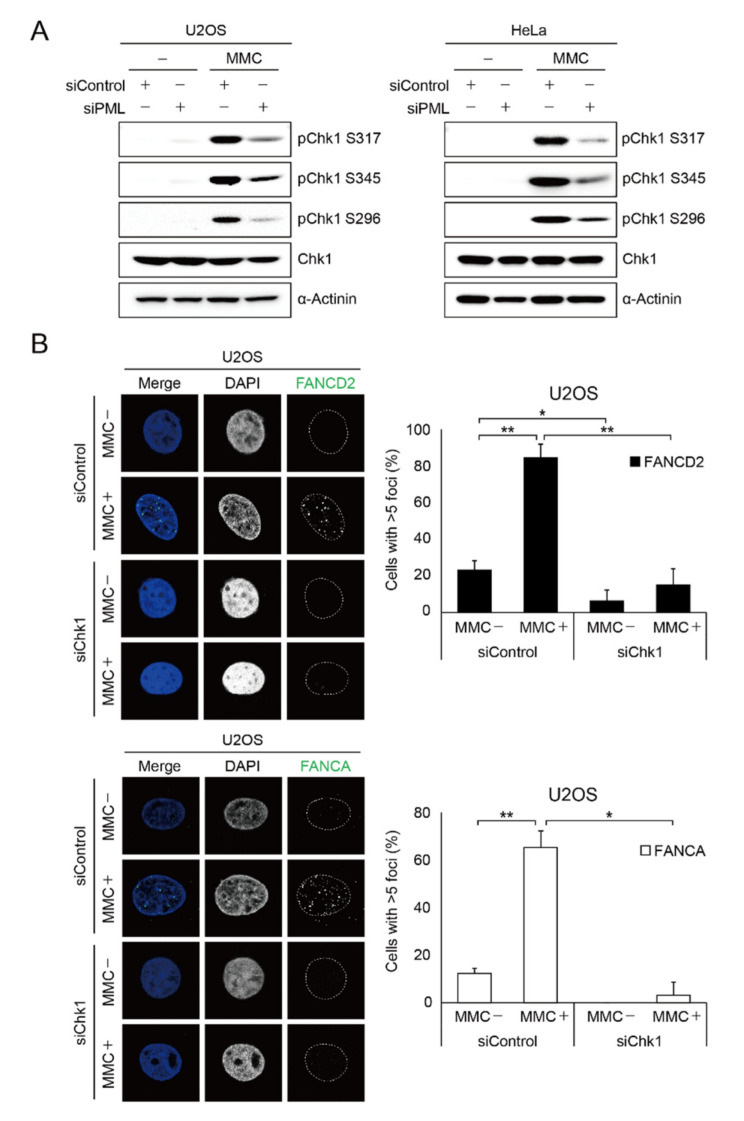
Depletion of *PML* impairs phosphorylation of CHK1 in response to DNA damage. (**A**) Phosphorylation of CHK1 in U2OS and HeLa cells treated with si*PML* in the absence or presence of 1 μM MMC. (**B**) U2OS cells treated with CHK1 siRNA were immunostained by anti-*FANCD2* or anti-*FANCA* antibody in the absence or presence of 1 μM MMC exposure. Representative immunofluorescence images and their quantification are shown. Over 37 nuclei were counted for statistics. Data represent the mean ± SD from three independent experiments (** *p* < 0.01; * *p* < 0.05).

The next question to be addressed is to understand the molecular basis of the transcriptional regulation of FA proteins upon *PML* depletion. It was reported that depletion of *PML* results in impairment of homologous recombination pathways and defects in damage-induced CHK1 S317 phosphorylation [12]. CHK1 is one of the important mediators of DNA damage responses and phosphorylates diverse downstream effectors implicated not only in the cell cycle, DNA repair, and replication, but also in gene expression [28]. Therefore, we tested if *PML* regulates damage-induced CHK1 phosphorylation. To this end, we depleted *PML* in the ALT cell, U2OS, and non-ALT cells, HeLa, and performed immunoblot analysis for damage-induced CHK1 phosphorylation. As shown in the Figure 4A, we found that ATR-mediated phosphorylation at Ser317 and Ser345, and autophosphorylation at Ser296 of CHK1, were significantly reduced in both U2OS and HeLa cells. These findings suggest that *PML* might promote FA gene expression and damage-induced foci formation via CHK1 phosphorylation. Indeed, the immunofluorescence experiment showed that damage-induced *FANCD2* and *FANCA* foci formation is severely impaired in U2OS cells treated with CHK1 siRNA (Figure 4B).

### 2.5. CHK1 Is Implicated in FA Gene Expression

**Figure 5:**
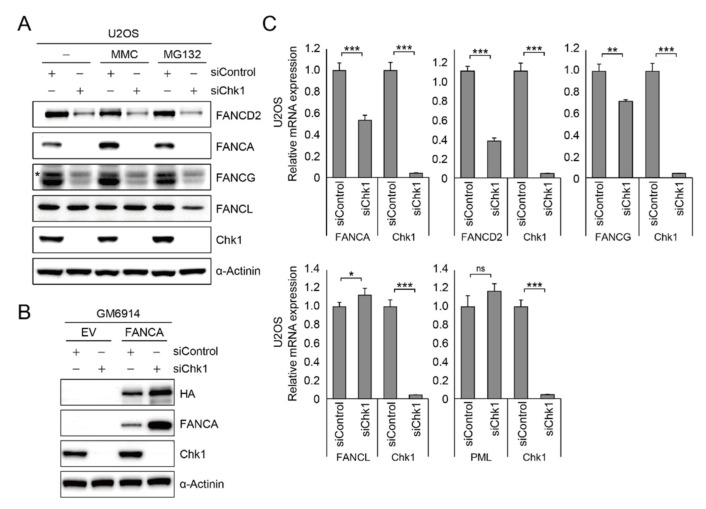
CHK1 is required for the transcriptional regulation of Fanconi anemia core protein and *FANCD2*. (**A**) U2OS cells were transfected with siControl or siCHK1 and treated with 1 μM mitomycin C (MMC) or 10 μM MG132 for 24 h. Protein expressions were determined from whole cell lysates using antibodies against *FANCD2*, *FANCA*, *FANCG*, *FANCL*, and CHK1. The asterisk indicates a cross-reactive artifact. (**B**) After depletion of CHK1, lysates prepared from GM6914/*FANCA* cell lines were subjected to immunoblot analysis with HA, *FANCA*, and CHK1 antibodies. (**C**) After depletion of CHK1, relative mRNA levels of core FA genes, *FANCD2*, and *CHK1* were assessed through RT-qPCR assay in U2OS cells. Data represent the mean ± SD from three independent experiments (*** *p* < 0.001; ** *p* < 0.01; * *p* < 0.05; ns, not significant (*p* > 0.05)).

To this point, we showed that CHK1 phosphorylation is regulated by *PML*. To test if CHK1 regulates gene expression, we depleted CHK1 in U2OS cells and performed immunoblot analysis to measure the expression of FA proteins. As shown in the Figure 5A, siRNA-mediated CHK1 depletion results in significant reduction in *FANCA* and decreased protein expression of *FANCD2* and *FANCG* (Figure 5A and Supplementary Figure S2B). However, as was the case with *PML* knockdown, *FANCL* protein expression was not affected by CHK1 depletion, which suggests that CHK1 does not regulate all FA protein expression. Again, we found that treatment with the proteasome inhibitor MG132 failed to restore the expression level of those proteins, excluding the possibility of ubiquitin-dependent proteasomal degradation of those proteins (Figure 5A). In addition, the *FANCA* expressed under CMV promoter in GM6914 cells did not decrease in cells depleted with CHK1, demonstrating that CHK1 regulates gene expression of *FANCA*, *FANCG*, and *FANCD2* through the endogenous promoter region (Figure 5B). As CHK1 depletion or treatment with a CHK1 inhibitor did not affect cell cycle progression (Supplementary Figure S3), reduced protein expression might not be due to aberrant cell cycle distribution. These findings suggest that CHK1 is implicated in transcription of *FANCA*, *FANCG*, and *FANCD2*. Next, to directly measure the transcriptional activity of *FANCA*, *FANCG*, and *FANCD2*, CHK1 was depleted in the U2OS cells and we performed RT-qPCR. As shown in the Figure 5C, we found that *FANCA*, *FANCG*, and *FANCD2* transcripts were severely decreased in the cells depleted with CHK1, which is consistent with the decreased proteins expression. However, *FANCL* transcript level was not altered in the same setting, indicating that not all transcription of the Fanconi core complex was not affected by CHK1 phosphorylation.

### 2.6. Synergistic Chemotherapeutic Effects of CHK1 Inhibitors and Crossliking Agents

**Figure 6:**
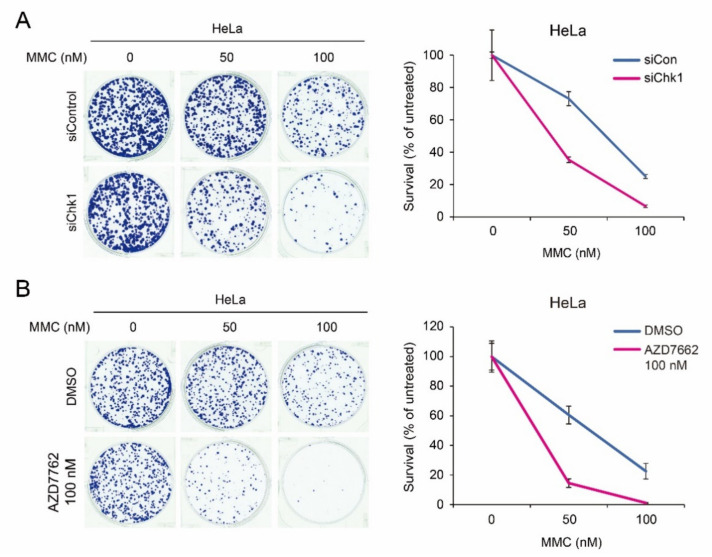
Depletion of CHK1 results in enhanced sensitivity to MMC, which induces ICLs. (**A**) Sensitivity of MMC was determined by clonogenic assay in HeLa cells treated with siControl or siCHK1. Fixed number of cells were seeded and after 24 h of MMC treatment at the final concentration of 0 to 100 nM. After one week, the colonies were stained by ultraviolet solution. (**B**) Chemotherapeutic efficacy of HeLa cells treated with 100 nM AZD7762 after 24 h of treatment with indicated concentration of MMC. After one week, the colonies were stained by ultraviolet solution.

DNA crosslinking agents have been used as chemotherapeutics for decades because they cause deadly DNA damage. The FA pathway is implicated in repairing DNA damage induced by DNA crosslinkers. Our findings showed that CHK1 phosphorylation plays a role in expression of some of the Fanconi anemia core factors and *FANCD2*, which is critical for ICL repair. Therefore, it is possible that depletion of CHK1 might be able to enhance the chemotherapeutic efficacy when combined with DNA crosslinking agents. To test this possibility, we depleted CHK1 for four days, plated a fixed number of the cells with different concentrations of MMC, and performed a clonogenic assay. As shown in the Figure 6A, we found that CHK1 depletion further sensitized the HeLa cells to MMC, implying that combined treatment with DNA crosslinking agents and CHK1 inhibitors might exhibit synergistic effects on cancer cell death. Indeed, combined treatment of CHK1 inhibitor AZD7762 and MMC led to enhanced cell death (Figure 6B).

## 3. Discussion

In this study, we demonstrated that *PML* regulates damage-induced CHK1 phosphorylation, which is important for *FANCA*, *FANCG*, and *FANCD2* gene expression at the transcriptional level. Fanconi anemia proteins are critical for repairing interstrand DNA crosslinks, which are generated by chemotherapeutic agents including mitomycin C, cisplatin, oxaliplatin, and carboplatin. Our findings suggest that defects in CHK1 phosphorylation result in the decrement of gene expression of some of the Fanconi anemia genes, which leads to failure of repairing interstrand DNA crosslinks. Indeed, we found that depletion of CHK1 or inhibition of CHK1 phosphorylation further sensitized HeLa cells to MMC (Figure 6). Since CHK1 inhibitors are one of the most investigated drugs in clinical trials, our findings will be informative to understand the molecular basis of the synergistic chemotherapeutic effects of CHK1 inhibitors and crosslinking agents.

*PML* proteins are essential for the formation of the distinctive subnuclear structure named *PML* NB, which plays diverse roles in transcriptional regulation, DNA damage responses, DNA repair, and cell proliferation [6,34]. We found that damage-induced CHK1 phosphorylation is diminished in cells depleted with *PML* expression in both ALT and non-ALT cells, although subcellular localization of *PML* NBs is different [34]. It was reported that ATR, the primary CHK1 kinase, is co-localized with ALT-associated *PML* NBs [36]. Therefore, it is possible that ALT-associated *PML* NBs are the center for CHK1 phosphorylation in response to DNA damage in ALT cells. This hypothesis is supported by our results showing that depletion of *PML* leads to significantly reduced damage-induced CHK1 phosphorylation at Ser317, Ser345, and Ser296 (Figure 4A). However, as ATR is not co-localized with TRF1 in non-ALT cells [36], *PML*-mediated CHK1 phosphorylation in non-ALT cells might be regulated differently, which remains elusive. Considering the facts that CHK1 plays a central role in DNA damage responses, further studies will be required for determining how *PML* regulates CHK1 phosphorylation in ALT and non-ALT cells.

Fanconi anemia is a rare recessive genetic disorder characterized by congenital abnormality, bone marrow failure, and cancer predisposition. Genome instability has been observed in the cells that lost one of the factors implicated in the Fanconi anemia pathway. Although FA is a rare genetic disease, factors involved in FA pathways have been extensively studied as they are associated with important DNA repair factors and pathways. Nevertheless, transcriptional regulation of FA gene expression has not been investigated intensively. Here, we found that *FANCA*, *FANCG*, and *FANCD2* transcription is downregulated in the cells depleted with CHK1 (Figure 5). It was reported that some of the FA gene expression was regulated by the Rb/E2F pathway [37]. Further studies showed that the endogenous *FANCD2* promoter interacts with the E2F transcriptional activator [37]. In addition, recent study showed that CHK1 phosphorylation or CHK1 depletion leads to downregulation of *E2F1* [29]. Therefore, we propose that damage-induced CHK1 phosphorylation promotes *E2F1* gene expression, which activates transcription of some of the FA genes.

In conclusion, we demonstrated that *PML* regulates damage-induced CHK1 phosphorylation, which promotes *FANCA*, *FANCG*, and *FANCD2* gene expression. Cancer cells with p53 mutations rely heavily on CHK1 functions for cancer cell survival, which makes CHK1 a target for cancer therapy. Indeed, the enhanced chemotherapeutic effects of CHK1 inhibitors have been reported. Therefore, CHK1 inhibitors are often used with platinum-based drugs or gemcitabine. Specifically, cancer stem cells exhibit more efficient DNA repair systems [38], which often leads to resistance to chemotherapy. Here, we showed for the first time that CHK1 is implicated in the expression of FA proteins, which is important for repairing interstrand DNA crosslinks. These findings suggest that treatment with CHK1 inhibitors might be able to reduce the DNA repair capacities of cancer cells, which increases the cancer therapeutic efficacy.

## 4. Materials and Methods

### 4.1. Cell Culture

U2OS, HeLa, and GM6914 cells were cultured in Dulbecco’s Modified Eagle Medium supplemented with 10% fetal bovine serum and 100 U/mL penicillin and streptomycin (all Gibco, Grand Island, NY, USA). All cells were maintained in humidified 5% CO_2_ atmosphere at 37 °C.

### 4.2. Small Interfering RNA (siRNA)

*PML*, ATR, CHK1, or control siRNA was transfected into cells using Lipofectamine RNAiMAX reagent (Invitrogen, Carlsbad, CA, USA) as suggested by the manufacturer’s instructions. The siRNA sequences used in this experiment were as follows: siPML#1: 5‘-AACGACAGCCCAGAAGAGGAAUU-3′, siPML#2: 5′-CACCCGCAAGACCAACAACAUUU-3′, siATR#1: 5′-GGGAAAUACUAGAACCUCAUCUAAAUU-3′, siATR#2: 5′-GGUCUGGAGUAAAGAAGCCAAUUUAUU-3′, siATR#3: 5′-CCACCUGAGGGUAAGAACAUGUUAAUU-3′, siChk1#1: 5′-GAAGCAGUCGCAGUGAAGAUUGUAG-3′, siChk1#2: 5′-CAAGAUGUGUGGUACUUUACCAUAT-3′, siChk1#3: 5′-GAGAAGGCAAUAUCCAAUAUUUATT-3′; a pool of two si*PML*s, three siATRs, or three siChk1s were used with a final siRNA concentration of 25 nM.

### 4.3. Western Blotting

After 24 h from siRNA transfection, cells treated with 1μM MMC (Sigma, St. Louis, MO, USA) for 24 h were harvested by trypsinization and lysed by lysis buffer (50 mM Tris-HCl pH 7.5, 150 mM NaCl, and 0.5% NP-40) containing a protease inhibitor cocktail (Bio-Rad Laboratories, Hercules, CA, USA) and NanoDrop (Thermo Fisher Scientific, Waltham, MA, USA). Samples were eluted in protein sample buffer (Elpis Biotech, Taejon, Korea), boiled for 5 min, and quickly centrifuged. SDS-PAGE electrophoresis was conducted using 6% or 10% gels, and samples for *FANCD2* immunoblotting were loaded on a 10-well Novex 3~8% Tris-acetate gel (Invitrogen, Waltham, MA, USA). Proteins were transferred to a 0.45 μm pore immobilon-P transfer membrane (Merck Millipore, Burlington, MA, USA). Blots were blocked with 10% Difco^TM^ Skim Milk (BD Biosciences, East Rutherford, NJ, USA) for 1 h at room temperature and incubated with *FANCD2* (Novus Biologicals, Centennial, CO, USA: NB100-182), *FANCA* (Merck Millipore: MABC557), *FANCG* (Santa Cruz Biotechnology, Dallas, TX, USA: sc-393382), *FANCL* (Santa Cruz Biotechnology: sc-137067), HA (Covance, Princeton, NJ, USA: MMS-101R), CHK1 (Santa Cruz Biotechnology: sc-8408), pCHK1 S317 (Cell Signaling Technology, Danvers, MA, USA: #12302), pCHK1 S345 (Cell Signaling Technology: #2348), pCHK1 S296 (Cell Signaling Technology: #2349), Actinin (Santa Cruz Biotechnology: sc-166524), and Tubulin (AbFrontier, Seoul, Korea: LF-PA0146) as primary antibodies. After blots were washed in 1X PBS-Tween20 buffer, the blots were incubated in anti-mouse secondary (Jackson ImmunoResearch, West Grove, PA, USA: 115-035-003) or anti-rabbit secondary (Jackson ImmunoResearch: 111-035-003). Bands were detected by enhanced chemiluminescence solution (Bio-Rad Laboratories) using ChemiDoc System (Bio-Rad Laboratories).

### 4.4. Immunofluorescence

Cells were cultured on 12 mm diameter microscope cover glasses, permeabilized with PBS containing 0.1% Triton X-100 for 3 min, and then fixed with 3.7% formaldehyde (Sigma) for 10 min at room temperature. Cells washed by PBS were extracted with 0.5% NP-40 (USBiological, Salem, MA, USA) for 5 min at room temperature. After they were washed, cells were blocked with blocking buffer (0.2% gelatin and 0.5% BSA in PBS) for 1~2 h at room temperature and then incubated with primary antibodies, such as *FANCA* (Merck Millipore: MABC557), *FANCD2* (Novus Biologicals: NB100-182), *PML* (Santa Cruz Biotechnology: sc-966), or γH2AX (Cell Signaling Technology: #2595) overnight at 4 °C. Samples were washed three times for 10 min with blocking buffer. Secondary antibodies (Abcam, Cambridge, UK) mouse 488 (Abcam: ab150109), rabbit 594 (Invitrogen: A21207), mouse 594 (Abcam: ab150112), and rabbit 488 (Abcam: ab150061) were diluted 1:2500 and incubated for 1 h at room temperature in the dark. Finally, cells were washed three times for 30 min and mounted in Vectashield^®^ containing DAPI (Vector Laboratories, Burlingame, CA, USA).

### 4.5. Mitomycin C Sensitivity Assay

Cells were plated in 96-well plates in triplicate at a density of 1000 cells per well. MMC was added to cells treated with siRNA at final concentration from 0~100 nM. After 4 or 5 days in culture, cell numbers were determined using Cellvia Enhanced Cell Viability Assay Kit (AbFrontier). The cell number after MMC treatment was normalized to the cell number in the untreated sample to arrive at the percent survival.

### 4.6. RNA Isolation and Quantitative Real-Time PCR

Total cell RNA was extracted using RNeasy Mini Kit and QIAshredder (QIAGEN) and synthesized into cDNA through the SuperScript III First-Strand Synthesis System (Invitrogen); mRNA level was determined by 2× qPCRBIO SyGreen Blue Mix Lo-ROX (PCRBiosystems, Wayne, PA, USA), and relative value was calculated after normalization against GAPDH. Gene-specific primers are listed in Table S1.

### 4.7. Clonogenic Assay

Cells (5 × 10^5^/dish) were plated in a 6 cm culture dish for 1 day and transfected with 25 nM siRNA using RNAiMAX. The next day, cultured cells were treated with indicated MMC concentrations for 1 day, and then harvested cells (1 × 10^3^/well) were spread in new 6-well dishes. After 1 week, colonies were washed and fixed with 3.7% formaldehyde for 15 min at room temperature. Washed cells were stained with crystal violet solution (0.1% crystal violet and 10% ethanol in distilled water) for 15 min. After removing all dye, each plate was tapped on paper towels and air dried overnight.

### 4.8. Statistics

Statistics were calculated using GraphPad Prism software (San Diego, CA, USA). *p* values are presented as the mean ± standard deviation (SD). For comparison between two normally distributed test groups, the two-tailed unpaired Student’s t-test was used. The following standard symbols are used to reference *p* values: ns, not significant; * *p* < 0.05; ** *p* < 0.01; *** *p* < 0.001.

## Data Availability

Not applicable.

## Supplementary Materials

Supplementary materials can be found at https://www.mdpi.com/article/10.3390/ijms22157782/s1.
