# Supplementary material for: Promyelocytic Leukemia Proteins Regulate Fanconi Anemia Gene Expression
Source: Int J Mol Sci. 2021 Jul 21;22(15):7782. doi: 10.3390/ijms22157782 (PMC8346011; doi:10.3390/ijms22157782)

Supplementary Materials for

Article

Promyelocytic Leukemia Proteins Regulate Fanconi Anemia Gene Expression

Anudari Munkhjargal[1,†], Myung-Jin Kim[1,†], Dayeon Kim[1,†], Young-Jun Jeon[2], Younghoon Kee[3], Lark Kyun Kim[4,*] and Yonghwan Kim[1,*]

[1] Department of Biological Sciences & Research Institute of Women's Health, College of Natural Sciences, Sookmyung Women's University, Seoul 04310, Republic of Korea
[2] Department of Integrative Biotechnology, Sungkyunkwan University, Suwon 16419, Republic of Korea
[3] Department of New Biology, Daegu Gyeongbuk Institute of Science and Technology, Daegu 42988, Republic of Korea
[4] Severance Biomedical Science Institute, Graduate School of Medical Science, Brain Korea 21 Project, Gangnam Severance Hospital, Yonsei University College of Medicine, Seoul 06230, Republic of Korea
* Correspondence: lkkim@yuhs.ac (Tel.: +82-2-2019-5402) and yhkim@sookmyung.ac.kr (Tel.: +82-2-710-9552)
† These authors contributed equally to this work.

Supplementary Figure S1

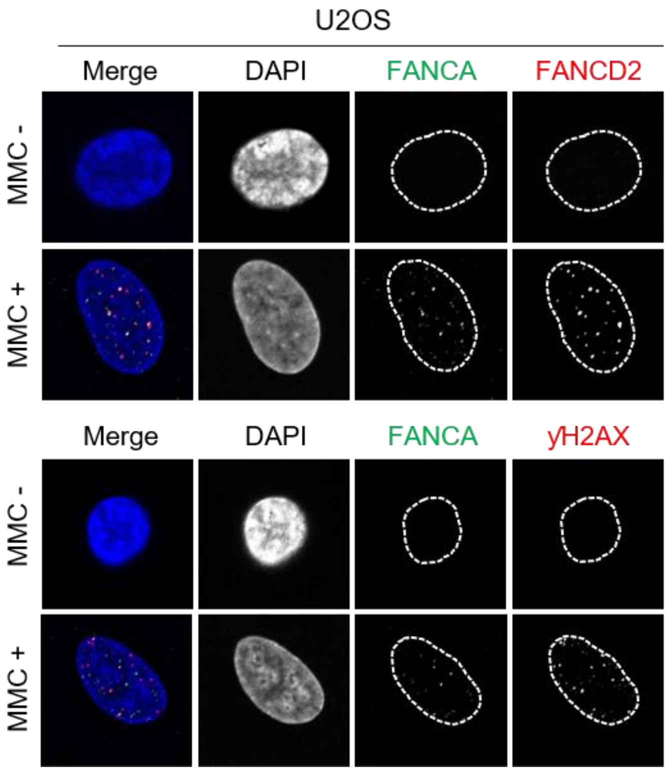

Figure S1. FANCD2 and FANCA recruited to DNA interstrand crosslinks induced by MMC. U2OS cells untreated or treated with 1μM mitomycin C for 24 hours were immunostained using anti-FANCA, anti-FANCD2, and γH2AX as primary antibodies and secondary antibody (green;FANCA, red;FANCD2, γH2AX). DAPI was used to stain the cell nuclei (blue). In merge images, co-localization of green and red foci displays yellow foci. Over 100 nuclei were observed, and the representative images were presented.

Supplementary Figure S2

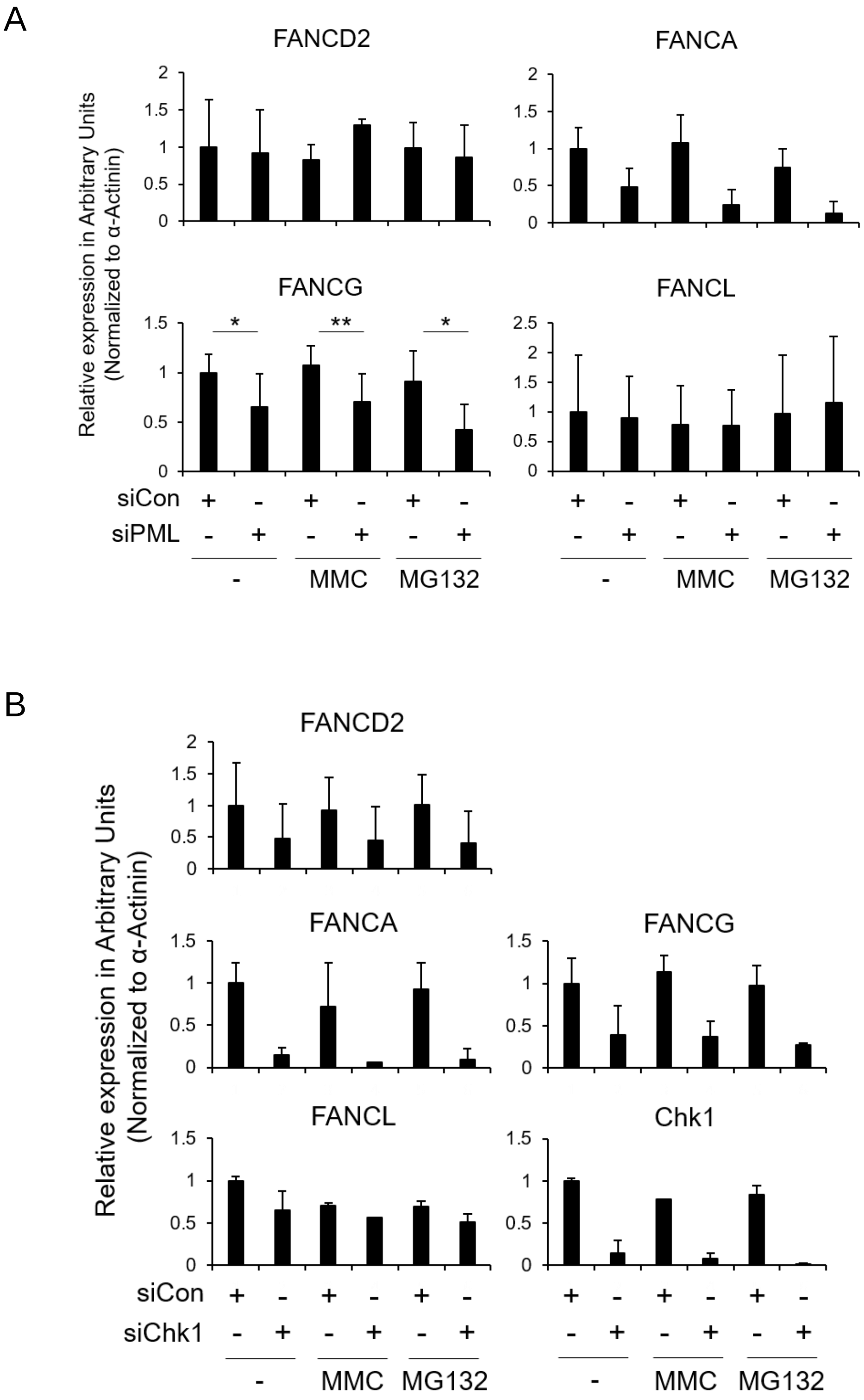

Figure S2. Quantified histograms of Figure 3A and 5A western blot. Each blot images in Figure 3A (N=4) **(A)** and Figure 5A (N=3) **(B)** are quantified by ImageJ software and the relative protein expression was indicated in the bar graph.

Supplementary Figure S3

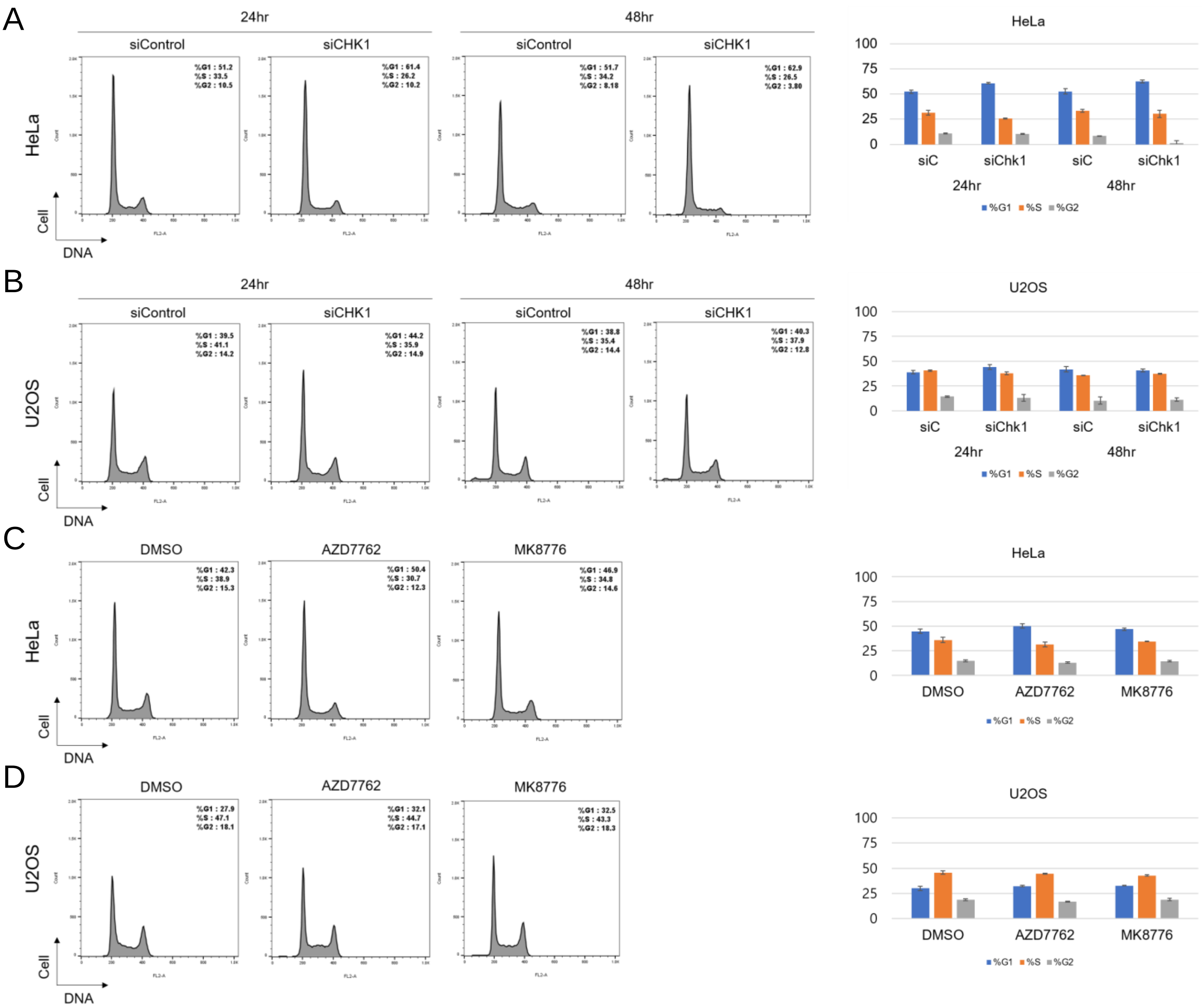

Figure S3. Cell cycle distribution under condition of siChk1 or Chk1 inhibitors. In HeLa **(A)** and U2OS **(B)** depleted with siChk1 for 24 h and 48 h, cell cycle distribution were explored by FACS analysis. Cell cycle proportion of HeLa **(C)** and U2OS **(D)** treated with 100 nM AZD7762 or 500 nM MK8776 for 24 h were analyzed by FACS.

Supplementary Table S1

Sample		Sequence 5' → 3'
FANCD2	Sense	GCCGACTGAAACAGGGAGAA
	Antisense	GAGCACACTGGAAACTGGGA
FANCA	Sense	CCGTGGGTATTCTCTCAGCC
	Antisense	GGAAGACAGCTTCTTTCTCTGC
FANCG	Sense	ATACTAGCAAGCAGGGCAGG
	Antisense	CTTGGCTCCGAGCTATCCAG
FANCL	Sense	TGGACACCTCAGAGCTCCTT
	Antisense	TGCACTCCGTGGAGGTTTTT
PML	Sense	CTGTGGTACAGTCAGTGCCC
	Antisense	CTCTTCTGGGCTGTCGTTGT
Chk1	Sense	TGGTTGACTTCCGGCTTTCT
	Antisense	AAACCTTCTGGCTGCTCACA
GAPDH	Sense	GCAAATTCCATGGCACCGTC
	Antisense	TCGCCCCACTTGATTTTGGA

Table S1. qPCR primer sequences.

Unprocessed blot

Figure 1A

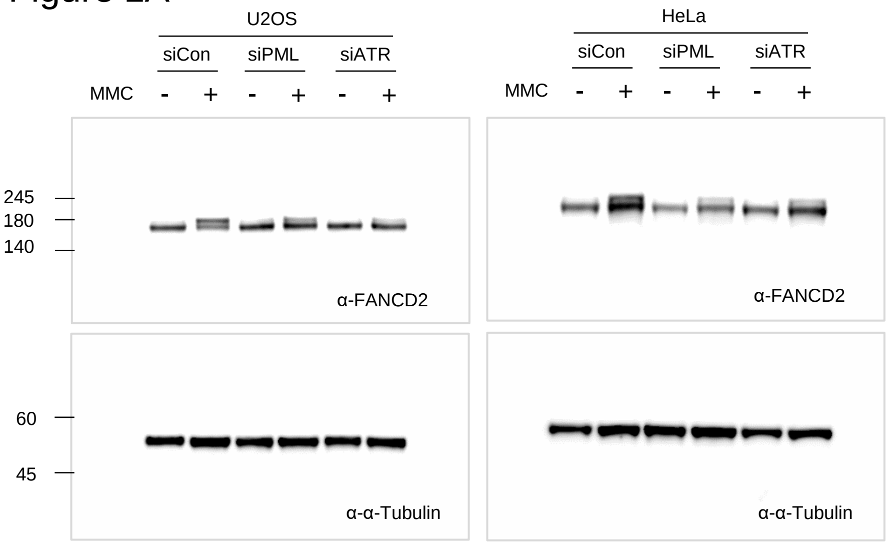

Figure 2B

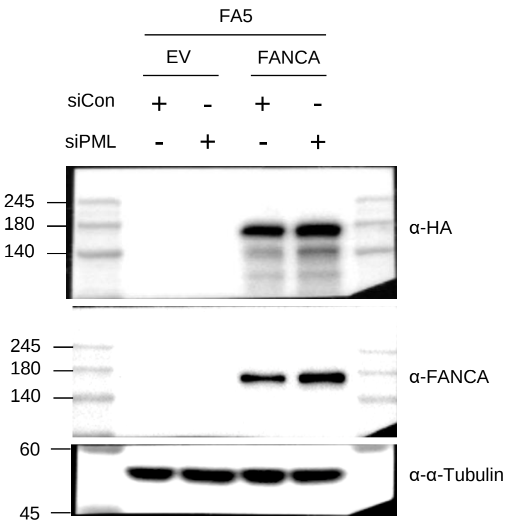

Figure 3A

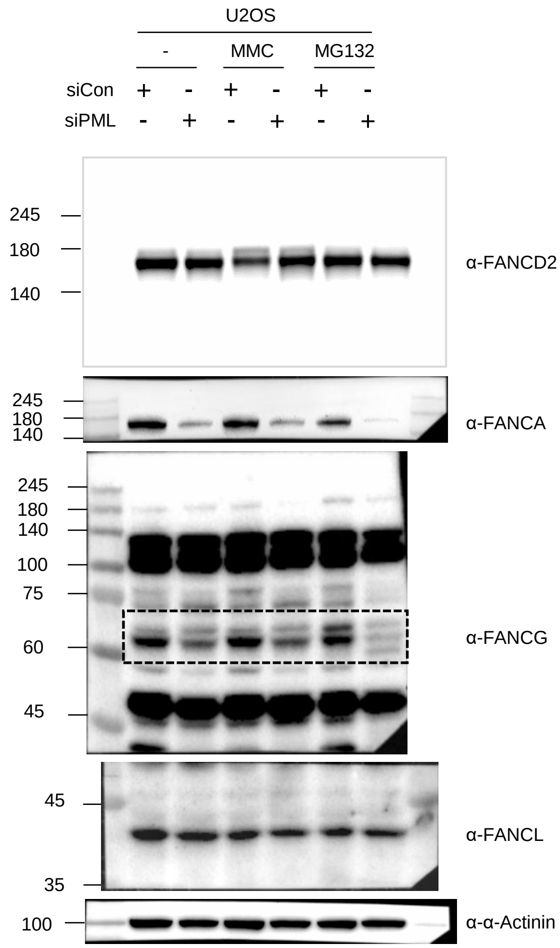

Figure 4A

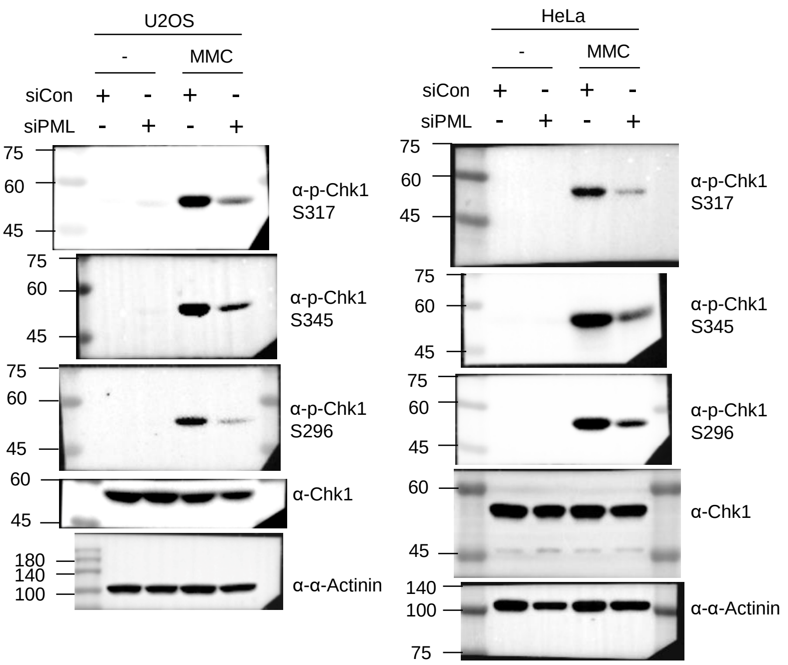

Unprocessed blot

Figure 5A

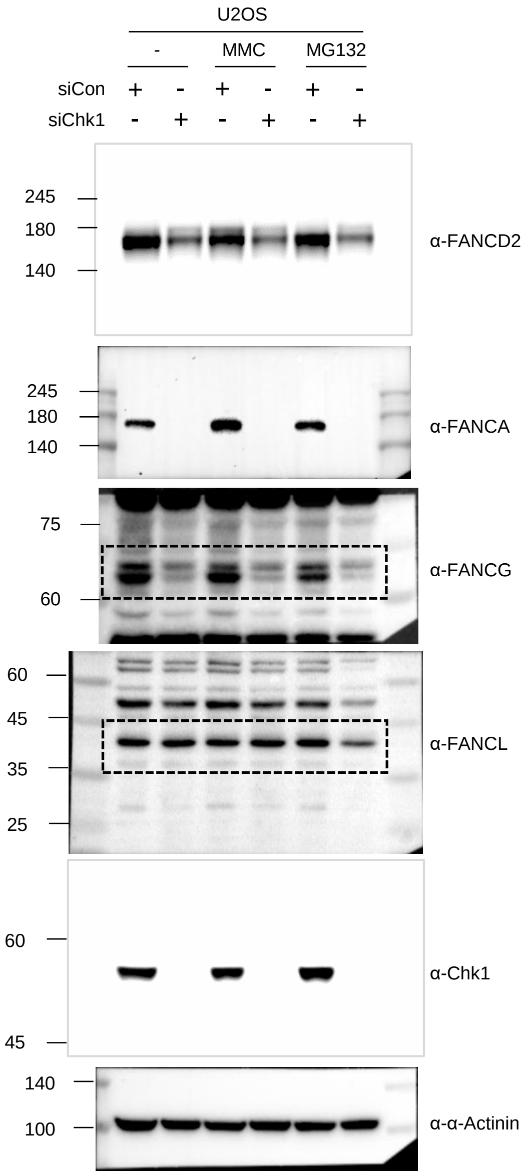

Figure 5B

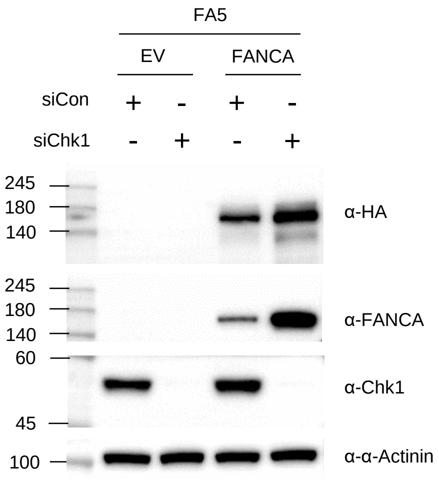

Supplement: Supplementary file 1 [file ijms-22-07782-s001.zip › ijms-1177720-supplementary.pdf]
